# Removal of Carmine from Aqueous Solution by Carbonated Hydroxyapatite Nanorods

**DOI:** 10.3390/nano7060137

**Published:** 2017-06-05

**Authors:** Guanxiong Liu, Caibao Xue, Peizhi Zhu

**Affiliations:** School of Chemistry and Chemical Engineering, Yangzhou University, Yangzhou 225002, China; orthopeadicsman@163.com (G.L.); xcb308999280@163.com (C.X.)

**Keywords:** carbonated hydroxyapatite, nanorods, carmine, water treatment

## Abstract

In this study, carbonated hydroxyapatite (CHA) nanorods were prepared by a novel hydrothermal method. The crystallinity and chemical structure of synthesized CHA nanorods was characterized by transmission electron microscopy (TEM), X-ray diffraction (XRD), Raman spectroscopy and X-ray photoelectron spectroscopy (XPS), respectively. Carmine was selected as representative organic dyes to study the adsorption capacities of CHA nanorods. Mechanistic studies of carmine adsorption by CHA nanorods show that the adsorption processes both follow the pseudo-second-order kinetic model and fit the Langmuir isotherm model well. The CHA nanorods exhibited a high adsorption capacity of 85.51 mg/g for carmine at room-temperature. The experimental results prove that CHA nanorods can be promising absorbents for removing organic dye pollutants in wastewater from paper and textile plants.

## 1. Introduction

Organic dyes widely exist in the effluent from paper and textile industrial wastewater and can cause serious environmental problems and damages to human health [1,2]. Over recent years, hydroxyapatite (HA) and its composites have been extensively studied for its applications in removing heavy metal ions such as Pb^2+^, Hg^2+^, Cd^2+^ and ampicillin in wastewater treatment [3,4,5]. Because of its excellent adsorption effect, HA has been used to remove metal ions and organic compounds from industrial wastewater. The carbonate hydroxyapatite (CHA) is a form of HA in which OH^−^ ions or PO_4_^3−^ ions in apatite lattice are partially substituted by carbonate ions. Doping HA with carbonate ions can effectively enhance the absorption efficiency by increasing specific surface areas and active sites on an apatite surface [6].

Since the absorption efficacy of HA can be influenced by tailoring morphology and surface structures of apatite, synthesizing HA nanoparticles with unique morphologies have attracted great interest in this field [7,8,9,10,11,12,13,14,15,16,17,18,19]. Recently, doping different ions in apatite lattice has been proved to be an effective way to enhance the absorption efficiency of HA [8]. Several templates including polydopamine [9], carbon spheres [10], and anodic aluminum oxide (AAO) [11], polyvinyl pyrrolidone (PVP) [12], polyethylene glycol (PEG) [13], and Ethylene Diamine Tetraacetic Acid (EDTA) [14] have been used to synthesize HA nanoparticles with different crystal shape and surface morphologies.

In recent years, HA nanorods have attracted a lot of attention due to their unique rod structure and large specific surface area [15,16]. While the use of common HA powder for removing pollutants has been extensively studied, there are seldom reports about adsorption of dyes by HA nanorods. Doping HA nanorods with carbonate ions could also possibly enhance the absorption efficiency. Therefore, in this study, we prepared highly crystalline CHA nanorods by using the Ethylene Diamine Tetraacetic Acid (EDTA) and Cetyltrimethyl Ammonium Bromide (CTAB) as a template through the hydrothermal method. The obtained CHA nanorods were investigated by High Resolution Transmission Electron Microscopy (HRTEM), X-ray diffraction (XRD), Raman, X-ray photoelectron spectroscopy (XPS) and Brunauer-Emmett-Teller (BET) analysis. Carmine was selected as representative organic dyes to test the adsorption capacities of CHA nanorods. The factors influencing carmine adsorption by CHA nanorods such as contact time, solution pH value, and initial concentration were systematically studied. The removing rates and adsorption mechanism of carmine by CHA nanorods were further investigated by the kinetics models and the adsorption isotherm models, respectively. The goal of the study is to develop a promising CHA adsorbent with high adsorption capacity for organic dye pollutants.

## 2. Materials and Methods

### 2.1. Materials

Ca(NO_3_)_2_·4H_2_O (AR, Sinopharm Chemical Reagent, Shanghai, China), (NH_4_)_2_HPO_4_ (AR, Sinopharm Chemical Reagent, Shanghai, China), CTAB (AR, Sinopharm Chemical Reagent, Shanghai, China), and EDTA (AR, Sinopharm Chemical Reagent, Shanghai, China) were used as received without further purification.

### 2.2. Samples Synthesis

In the typical hydrothermal synthesis of CHA nanorods, Ca(NO_3_)_2_·4H_2_O and (NH_4_)_2_HPO_4_ were used as calcium source and phosphorus source, respectively. EDTA and CTAB served as templates for CHA nanorods. All the chemicals were used as received without further purification. 7.887 g of Ca(NO_3_)_2_·4H_2_O, 5.7 g of EDTA and 1 g of CTAB was dissolved in 30 mL deionized water with magnetic stirring, then 0.2772 g of NH_4_HCO_3_ and 2.6412 g of (NH_4_)_2_HPO_4_ was dissolved in 20 mL deionized water. The phosphorus and carbonate source solution was added dropwise to calcium solution, meanwhile keeping pH about 9~11 by adding ammonium hydroxide solution. After 5 min stirring, the hydroxyapatite suspensions were poured into Teflon-lined stainless steel autoclaves. The autoclaves were placed in an oven for 24 h at 180 °C for hydrothermal synthesis. When the hydrothermal reaction was completed, the autoclave was cooled down to room temperature. Then the precipitate was washed by deionized water and ethyl alcohol three times, and dried for 6 h at 80 °C.

### 2.3. Characterization Methods

CHA nanorods were examined by transmission electron microscope (TEM, Tecnai 12, Philips, Amsterdam, Holland) and selected electron diffraction (SEAD, Tecnai 12, Philips, Amsterdam, Holland) for structural characterization. The size distribution of nanoparticles from TEM micrographs was measured by Nano measurer software (1.2). In total, 88 points were selected to analyze the size distribution of HA nanorods. The molecular structure of CHA was analyzed by Raman spectroscopy (DXR, GX-PT-2412, Thermo, Waltham, MA, USA) with 532 nm laser as excitation wavelength. XRD spectrum was recorded on an X-ray powder diffractometer using CuKα radiation operating at 40 kV and 30 mA (XRD, D8 ADVANCE, Bruker-AXS, Bremen, Germany). The elements composition of the sample was analyzed by X-ray photo-electronic spectroscopy (XPS, ESCALAB250Xi, Thermo Fisher Scientific, Waltham, MA, USA), using a monochromated Al Kα X-ray source. The samples were scanned at a reflection angle (2θ) using a step rate of 1.0 deg/min. Fourier transform infrared spectrometry (FTIR, ALPHA, Bruker, Blaireka, MA, USA) was used to identify the molecular structure of CHA nanorods. FTIR spectrum of CHA nanorods was recorded from 500 to 3600 cm^−1^. The surface and porosity of as-prepared CHA nanorods were characterized by Brunauer-Emmett-Teller (BET) analyzer (ASAP 2020 HD88, Micromeritics, Norcross, GA, USA).

### 2.4. Carmine Adsorption Experiments

The potential activities of CHA nanorods for pollutants adsorbing were investigated by carmine adsorption. The contact time, the pH value of the solution and the initial pollutants concentrations were studied as important factors that affect the adsorption activities during experiments.

The adsorption experiments of carmine were performed by bath methods. The CHA nanorods were immersed into prepared solution under different adsorption time, pH value and initial concentrations. The CHA nanorods were separated from supernatant by centrifugation at 4000 rpm for 10 min when adsorption equilibrium was achieved. The residual carmine in solution was analyzed using a Ultraviolet–visible spectroscopy (UV-Vis) spectroscopy at λ = 521 nm. All measurements were carried out at room temperature. In order to determine the adsorption capacities (*q_e_*, mg/g) of CHA nanorods for carmine, the *q_e_* was calculated according to the following formula:

(1) $$q_{e}=\frac{\left(C_{0}-C_{e}\right)V}{m}$$

where *C*_0_ and *C_e_* are the initial and final pollutants concentrations (mg/L), while *m* and *V* are the mass of CHA nanorods (g) and the volume of the solution (L), respectively.

## 3. Results and Discussion

### 3.1. Structural and Morphological Characteristics

**Figure 1:**
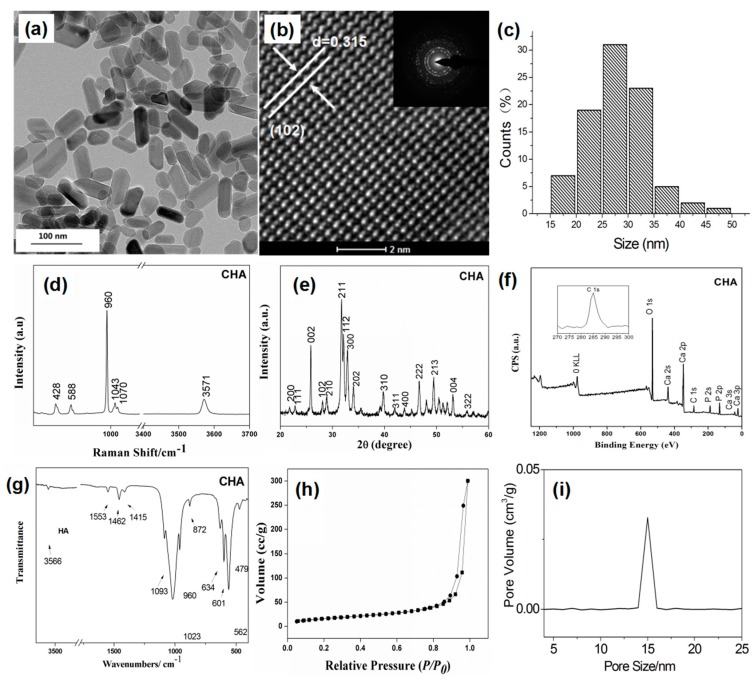
(**a**) Transmission electron microscopy (TEM) images; (**b**) selected electron diffraction (SEAD) pattern; (**c**) Size distribution; (**d**) Raman spectrum; (**e**) X-ray diffraction (XRD) pattern; (**f**) X-ray photoelectron spectroscopy (XPS) spectrum; (**g**) Fourier transform infrared spectrometry (FTIR) spectrum; (**h**) Brunauer-Emmett-Teller (BET) results and (**i**) Pore size distribution of synthesized carbonated hydroxyapatite (CHA) nanorods.

TEM was used to characterize morphologies and sizes of synthesized nanorods. Figure 1a–c shows TEM image, SEAD patterns and the size distribution of the synthesized CHA nanorods. Figure 1a shows that synthesized CHA nanorods have a length of about 60–90 nm. The SEAD patterns shows obvious multi-crystalline electron diffraction concentrate rings attributed to (002), (300), (310) and (211) crystallographic planes of hydroxyapatite [17,18]. As shown by Figure 1b, the HRTEM image of HA nanorods reveals a lattice spacing of 0.315 nm corresponding to the (102) crystal planes of HA, suggesting that the HA nanorod crystals predominantly grow along the *c*-axis direction [19] by diffusion-limited growth [20]. Figure 1c shows the size distribution of CHA nanorods which have an average width of 27.5 nm. Figure 1d shows the Raman spectrum of CHA nanorods. The OH^−^ peak was detected at 3571 cm^−1^. The peaks at 428 and 588 cm^−1^ were assigned to υ_2_ and υ_4_ mode of PO_4_^3−^, respectively. The strongest υ_1_ mode of PO_4_^3−^ appears at 960 cm^−1^ in the spectrum [21,22]. The peak at 1070 cm^−1^ should be attributed to the υ_1_ mode of B type CO_3_^2−^ substitution [23,24]. Figure 1e reveals XRD pattern of CHA nanorods. The peaks in XRD patterns can be assigned to the (200), (111), (002), (102), (210), (211), (112), (300), (202), (310), (311), (400), (222), (213), (004), and (322) crystallographic planes of hydroxyapatite in PDF 09-0432. The diffraction peaks of carbonated hydroxyapatite are slightly broader than the corresponding peaks of standard HA, indicating the decreased crystallinity of CHA nanorods when PO_4_^3−^ ions of hydroxyapatite crystal are partially substituted by carbonate ions. This result is in agreement with previous reports that substitution of CO_3_^2−^ in hydroxyapatite would cause lattice defects [25] and reduced crystal size and crystallinity in hydroxyapatite crystal [25,26]. The XPS spectra of CHA nanorods are shown in Figure 1f. One peak corresponding to C 1s was revealed at 285.1 eV, indicating that carbonate ions have been successfully incorporated into the apatite lattice structure. The carbonate content in CHA is measured as 1.54 wt %. Figure 1g shows the FTIR spectra of synthesized CHA nanorods. The broad and characteristic bands at 1023 and 562 cm^−1^ are assigned to the PO_4_^3−^ ions. Three peaks at 1093, 1023, and 960 cm^−1^ can be attributed to υ_1_ and υ_3_ phosphate modes, and 601 and 562 cm^−1^ are attributed to υ_4_ phosphate modes. The antisymmetric stretching vibration of C–O (υ_3_) in the region 1500–1400 cm^−1^ indicates that CO_3_^2−^ have been doped in synthesized nanorods. The υ_2_ vibration of CO_3_^2−^ at 872 cm^−1^ confirms the β-type substitution in CHA nanorods. Figure 1h shows the nitrogen adsorption of CHA nanorods and The BET results of obtained CHA nanorods were shown in Table 1. The adsorption isotherm of CHA shows typical type II behavior representing the mesoporous adsorption and the adsorption of nitrogen was observed at a relatively high pressure (*P*/*P*_0_ > 0.8). Figure 1i shows the pore size distribution of synthesized CHA nanorods. The average pore size of CHA nanorods examined by Barrett-Joyner-Halenda (BJH) method was about 14.9 nm. These mesopores with sizes ranging from 10 to 50 nm can provide high specific areas for adsorbed pollutants.

### 3.2. Carmine Adsorption Kinetics

#### 3.2.1. Adsorption Kinetics

**Figure 2:**
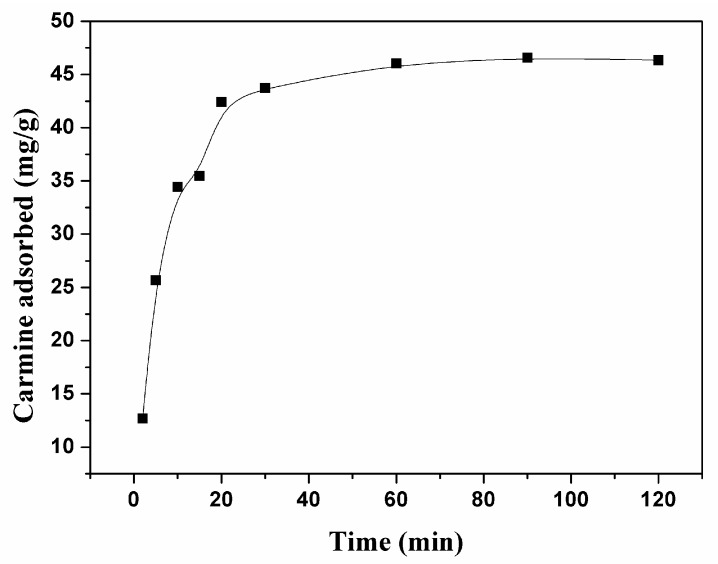
Adsorption activities of CHA nanorods for carmine with different contact times.

Figure 2 shows the adsorption capacity of carmine by CHA nanorods as a function of contact time. The high initial adsorption rate of carmine on CHA nanorods for the first 20 min indicates the quick removal of carmine molecules from aqueous solution, following by a slow kinetics to reach an equilibrium. The following pseudo-first-order and pseudo-second-order kinetic models were used for studying carmine adsorption kinetics [26,27].

Pseudo-first-order Equation:

(2) $$\ln\left(q_{e}-q_{t}\right)=\ln q_{e}-k_{t}t$$

Pseudo-second-order Equation:

(3) $$\frac{t}{q_{t}}=\frac{1}{k_{2}{q_{e}}^{2}}+\frac{1}{q_{e}}t$$

where *q_e_* and *q_t_* are the amounts of adsorbed carmine molecules (mg/g) on CHA nanorods at the equilibrium and at any time *t*, respectively. *k_t_* and *k*_2_ are the first order and the second order of rate constant of adsorption (g/mg min), respectively.

**Figure 3:**
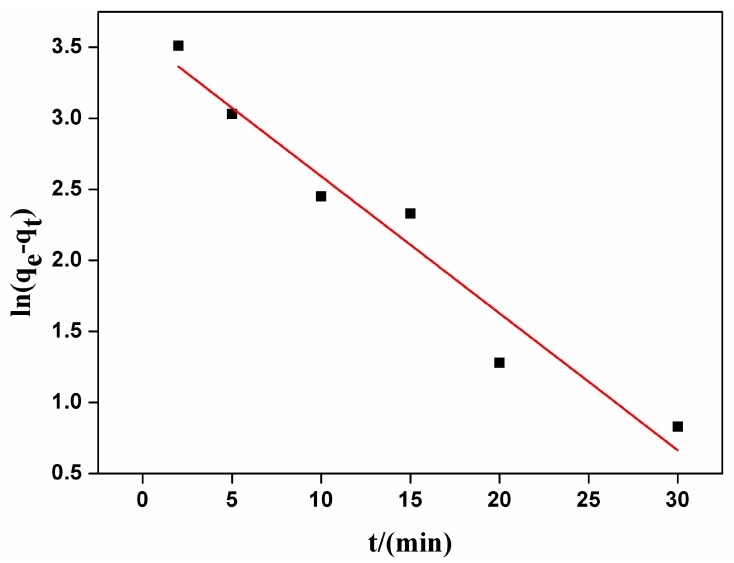
The pseudo-first order kinetics of carmine absorbed by CHA nanorods.

**Figure 4:**
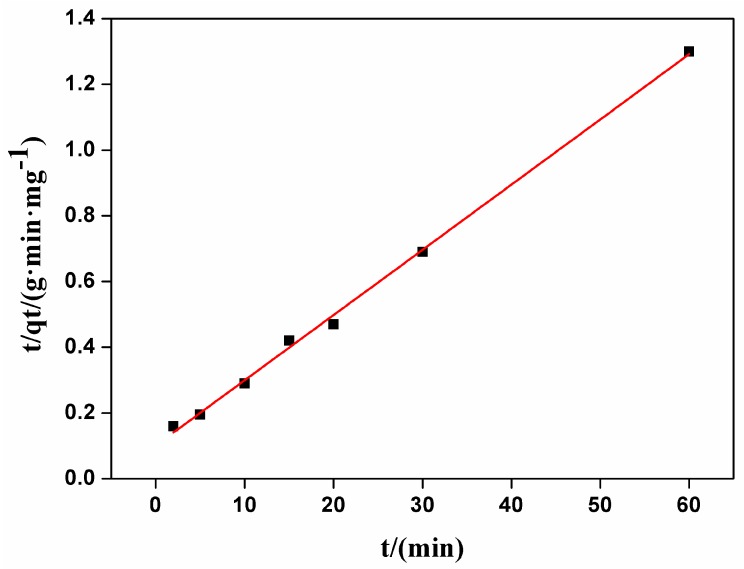
The pseudo-second order kinetics carmine absorbed on CHA nanorods.

These two types of kinetic curves were presented in Figure 3 and Figure 4. By comparing R^2^ of the dynamics models, the adsorption of carmine on CHA nanorods followed the pseudo-second order kinetic model better. As shown in Table 2, the experimental value (*q_e,exp_* = 46.57 mg/g) is closer to the value calculated by pseudo-second order model (*q*_2*e,cal*_ = 50.40 mg/g) than the value by pseudo-first order model (*q*_1*e,cal*_ = 35.02 mg/g). The well fitted linear plot of *t*/*q_t_* versus *t* with R^2^ = 0.9976 in Figure 4 proved that the adsorption process can be described by pseudo-second order kinetic model.

#### 3.2.2. Adsorption Isotherms

Adsorption studies with different initial concentration of carmine were performed in order to determine the CHA nanorods adsorption capacity and adsorption efficacy for carmine. The Freundlich and Langmuir adsorption isotherm models were used to fit the adsorption of carmine molecules onto CHA nanorods. The liner forms of Langmuir and Freundlich equation [27] can be expressed by following equations, respectively:

Freundlich model:

(4) $$\ln q_{e}=\ln K_{f}+\frac{1}{n}\ln C_{e}$$

where *q_e_* is the equilibrium sorption capacity (mg/g), *C_e_* is the equilibrium concentration of carmine molecules (mg/L), *K_f_* and *n* are the Freundlich constants and these constants are related to the adsorption capacity of the sorbent and the adsorption intensity.

Langmuir model:

(5) $$\frac{C_{e}}{q_{e}}=\frac{C_{e}}{q_{m}}+\frac{1}{K_{e}q_{m}}$$

where *q_e_* is the equilibrium adsorption capacity of carmine molecules (mg/g), *C_e_* is the equilibrium concentration of carmine molecules (mg/L), *q_m_* (mg/g) is the maximum sorption capacity, and *K_e_* (L/mg) is the Langmuir constant, which correlates to the energy of adsorption.

**Figure 5:**
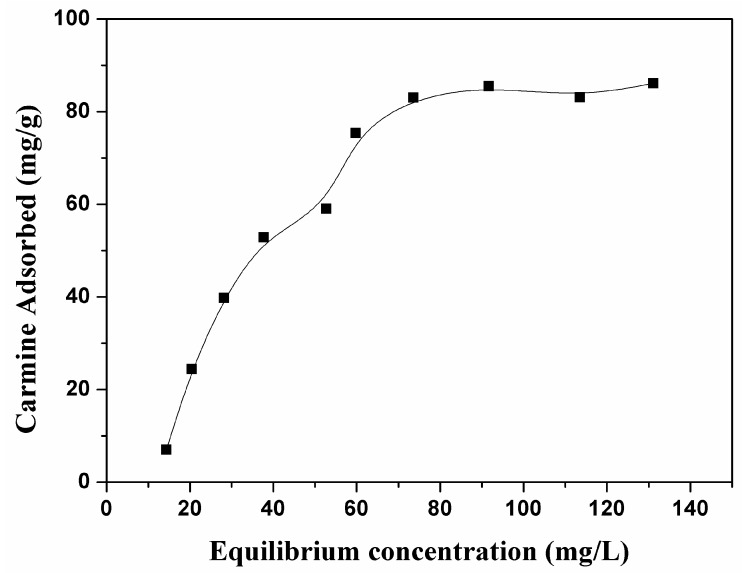
Adsorption activities of CHA nanorods for carmine with different initial concentrations.

**Figure 6:**
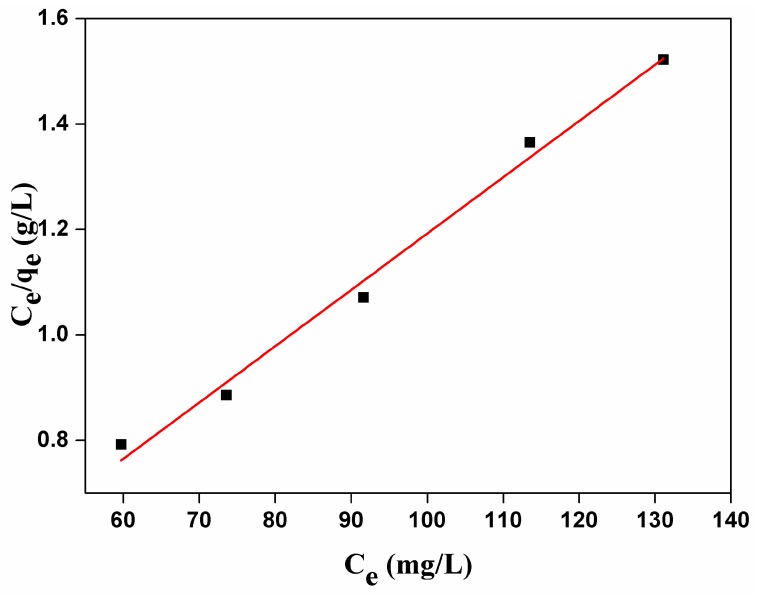
Langmuir adsorption isotherm for carmine absorbed by CHA nanorods.

**Figure 7:**
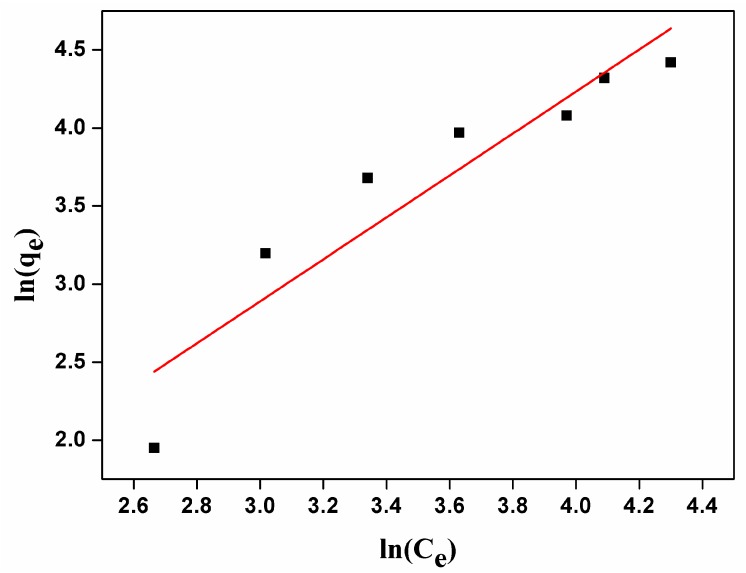
Freundlich adsorption isotherm for carmine absorbed by CHA nanorods.

As shown in Figure 5, the adsorption amount of carmine on CHA nanorods increased as the initial concentrations because higher concentration of carmine provides enough driving force for carmine molecules to diffuse onto the surface of CHA nanorods. The maximum adsorption capacities was 85.51 mg/g. As shown by Figure 6 and Table 3, the correlation coefficient R^2^ and constant n of Freundich adsorption isotherm model are 85.17 and 0.7439, respectively. Therefore, 1/*n* is not in the range of 0.1~1 and the adsorption process is not in good agreement with Freundlich model, indicating physical adsorption of carmine molecules on the surface CHA nanorods. As shown by Figure 7 and Table 3, fitting the experimental data using Langmuir isotherm gives a linear relationship with R^2^ = 98.867, suggesting a monolayer adsorption process of carmine molecules on the surface of CHA nanorods. The theoretical value of adsorption capacities, 93.63 mg/g, is close to the experimental data, 85.51 mg/g. The Langmuir constant *K_f_* was 0.0858 L/mg, implying a strong adsorption energy between carmine molecules and the surface of CHA nanorods. The binding of the carmine dye molecules on CHA surface is mediated by hydrogen bonding between the multi-hydroxyl groups of the dye molecules and PO_4_^3−^, OH^−^ and CO_3_^2−^ ions on the surface of CHA nanorods, and also the electrostatic interaction between positive carmine ionic form and negative surface charges of CHA nanorods.

## 4. Conclusions

In this study, we prepared carbonated hydroxyapatite (CHA) nanorods via hydrothermal synthesis method. CHA nanorods exhibited the adsorption capacity of 85.51 mg/g for carmine. The adsorption process of carmine fit well with the pseudo-second order kinetic model and Langmuir isotherm model. The adsorption experimental results show that the CHA nanorods have the potential properties for adsorption of organic dye pollutants from waste water.

## Figures and Tables

**Table 1:** Brunauer-Emmett-Teller (BET) results of carbonated hydroxyapatite (CHA) nanorods.

Sample Name	*S_BET_* (m^2^/g)	*V_P_* (cm^3^/g)	Pore Size (nm)
CHA	61.88	0.462	14.92

**Table 2:** Fitted kinetic parameters of carmine adsorption on CHA nanorods.

Kinetics Models	*q_e,exp_* (mg/g)	*q_e,cal_* (mg/g)	*k*_1_ (min^−1^)	*k*_2_ (mg/(g min))	R^2^
Pseudo-first-order model	46.57	35.02	0.095	-	0.9421
Pseudo-second-order model		50.40	-	0.00389	0.9977

**Table 3:** Kinetic parameters of Langmuir and Freundlich isotherms of carmine adsorption on CHA nanorods.

θ (*K*)	Langmuir			Freundlich		
	*q_m_* (mg/g)	*K_e_*	R^2^	*n*	*K_f_*	R^2^
298	93.63	0.0858	0.9887	0.7439	0.319	0.8517
